#  Glucose and Fluoxetine Induce Fine Structural Change in Human Serum Albumin 

**Published:** 2013

**Authors:** Minoo Shahani, Fatemeh Daneshi-Mehr, Roya Tadayon, Behrooz Hoseinzade Salavati, Ali-Reza Akbar Zadeh-Baghban, Abbas Zamanian, Mostafa Rezaei-Tavirani

**Affiliations:** a *Department of Base Science, Science and Research Branch, Islamic Azad University, Tehran, Iran.*; b *Proteomics Research Center, Shahid Beheshti University of Medical Sciences, Tehran, Iran. *; c *Faculty of Paramedical Sciences, Shahid Beheshti University of Medical Sciences, Tehran, Iran. *; d *Department of Surgery, Faculty of Medicines, Shahid Beheshti University of Medical Sciences, Tehran, Iran. *

**Keywords:** Human serum albumin, Glucose, Florescence, pH denaturation, Structural and functional alteration

## Abstract

Human serum albumin has been used as a model protein for protein folding and ligand binding studies over many decades. Due to its long life period and high concentration in plasma, HSA is highly sensitive to glycation. It is reported that 175 mg/dL glucose concentration is a threshold of kidney activity for the beginning of excretion of glucose. pH denaturation of HSA in absence and presence of different concentrations of glucose is studied and based on the Pace two-state model, the findings are analyzed. In addition, florescence emission data of albumin range in the period of 300-500 nm was depicted. The amounts of free energy change and [D]_1/2 _parameters of unfolding in correspond to florescence date indicate that glucose induces fine structural change in human serum albumin. Results showed that 175 mg/dL glucose concentration is a critical point for albumin structural and functional alteration.

## Introduction

The structure of human serum albumin has been determined by high-resolution X-ray crystallography (1). Human Serum Albumin (HSA), a single chain protein with 585 amino acids and molecular weight of ~ 67 KDa, is the most abundant protein in plasma which regards as a versatile protein with many important biological roles such as drug binding, regulation of plasma buffer, anti-oxidant function and anti-coagulant effect (2-4) and also in maintaining the pH and osmotic pressure of plasma (5). Human serum albumin has been used as a model protein for protein folding and ligand binding studies over many decades (6). Due to its long life period and high concentration in plasma, HSA is highly sensitive to glycation (7, 8) the most on Lys-525 and the less on sites of Lys-199, 281 and 489 *in-vivo *(9-12). The process of albumin glycation becomes very important especially in the case of diabetes where it can exert many adverse effects (13-16). In fact, many researchers believe that non-enzymatic glycation of albumin can alter its structure and function *e.g*., its binding properties, which in turn can leave many deleterious impacts in association with diabetes mellitus-related metabolic disorders (13). There are several evidences about conformational change of albumin in the presence of drugs such as Fluoxetine, CO-Amoxiclav and Acetaminophen (17-19).

The mechanism by which the proteins are folded from a structure-free denatured state to their unique biologically active state is an intricate process. This process is even more complex in multi-domain proteins where each domain may be able to refold independently and inter domain interactions may affect the overall folding process (20). HSA structure and dynamics are known to be influenced by a number of factors, like pH, temperature, and binding of different ligands (21). Elucidation of the mechanism of protein denaturation is important for understanding protein stability (22). HSA denaturation is described by a two-stage process. The first stage includes reversible structural alteration that is followed by irreversible structural alterations (6, 23). Here fine structural change of HSA in the presence of pathological concentration of glucose in corresponds to the clinical evidences is introduced and interpreted.

## Experimental

*Materials*

Human serum albumin (HSA) was purchased from Sigma chemical Co., USA, and used without defeating and the other substances of reagent grade were obtained from Merck chemical Co. In order to make HSA buffer, 0.7 mg/mL of HSA powder was dissolved in a 100 CC tris buffer (3.75 g Tris powder and 500 CC distilled water with pH of 7.5 by HCL) and pH was adjusted to 7.5 by adding distilled water. HSA buffer was incubated in 37°C serological water bath. Moreover, in order to make glucose sample, 0.5 g glucose was dissolved in 2 mL of distilled water, and for sampling the different concentration of glucose, different volumes of this sample were added to the HSA buffer (0, 50, 100, 150, 175 mg/dL).

*Denaturant buffer*

To denaturate the HSA, different amounts of HCl (pH= 0.9) from 0 up to 800 μL were added to the HSA buffer in absence and presence of different concentrations of glucose. The sample was incubated in 37°C serological water bath during 5 min.

*pH measurement*

**Figure 1:**
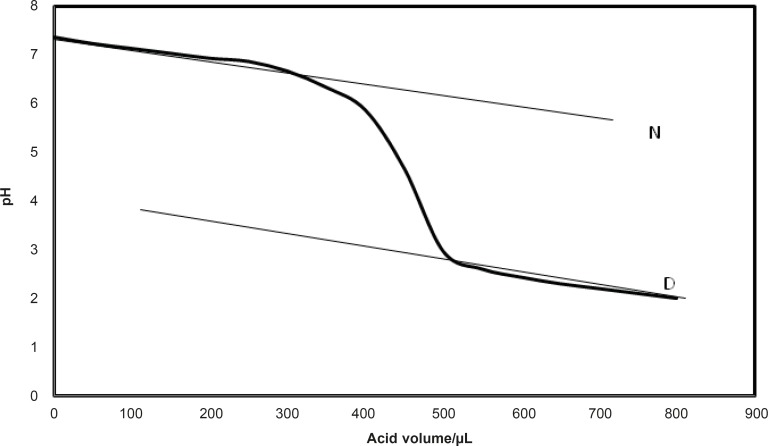
The native and denature lines that can be used for Pace analysis

The pH was measured by pH meter as it is described in detail in previous report (24). The native and denatured lines were defined for the obtained curves as it is shown in Figure 1. 

[D]_1/2_ as the volume of acid that causes 50% of denaturation (24), was calculated for all experiments. The experiments were repeated three times for each concentration of glucose and the average amounts of [D]_1/2_ was calculated. ΔG^0^_H2O_ as a main index of protein stability was described in the reported research (6). As a result, it is reported that fluoxetine induces conformational change in HSA structure (17). [D]_1/2_ was analyzed for albumin in the absence and presence of fluoxetine.

*Investigating albumin structural changes using florescence technique*

The fluorescence spectra of HSA are provided (100, 175 mg/dL) in the presence of glucose concentrations. HSA sample is exposed to 280 nm exposure after the incubation of glucose at 37°C for 5 min. Florescence emission rate of albumin range in the period of 300-500 nm was depicted and experiments were repeated three times.

## Results

**Figure 2:**
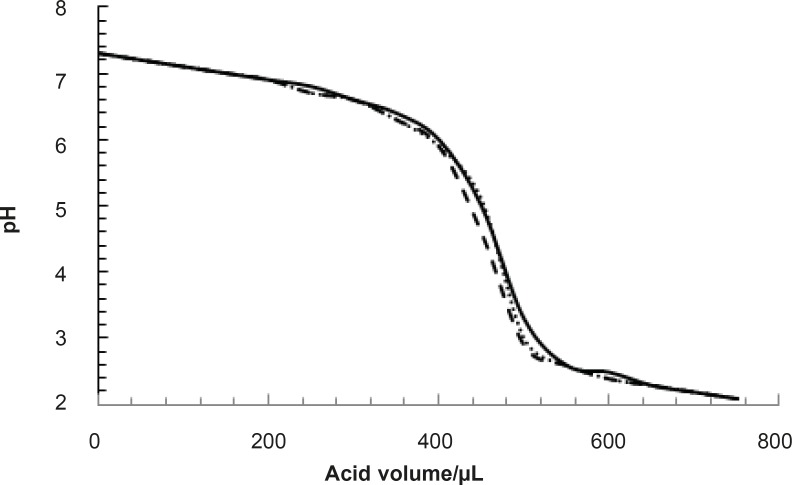
pH denaturation of albumin in the absence (zero concentration) of glucose, solid, dashed and pointed lines correspond to the three time repetition of experiment

pH denaturation of albumin was done in the presence of various concentrations of glucose. All experiments were repeated three times. One of such results is presented in Figure 2. 

**Figure 3:**
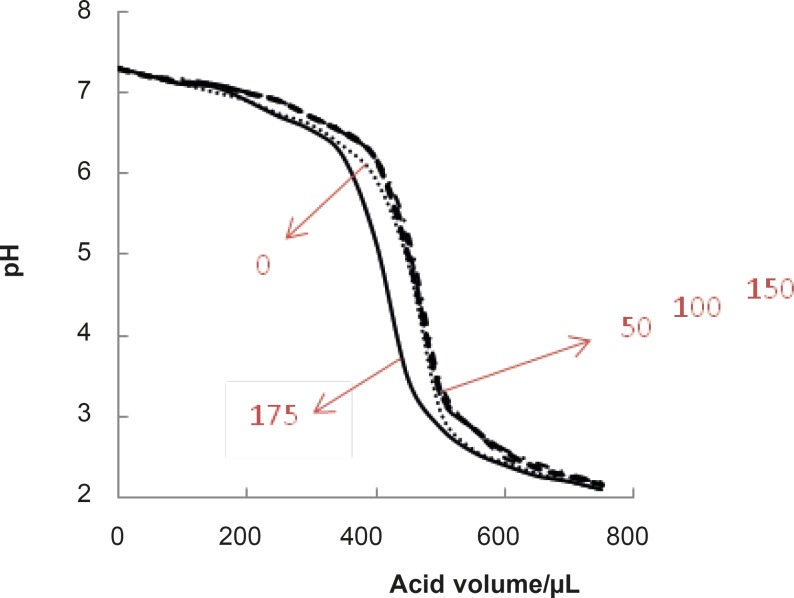
Mean curves of pH denaturation of albumin (mean of the three repetition curves for a certain concentration of glucose) in presence of the concentrations of glucose (0, 50, 100, 150, 175 mg/dL).

As it is reported, the normal range of blood glucose concentration is 100 mg/dL (7, 25). Therefore, this concentration and (0, 50, 150,175 mg/dL) concentrations of glucose were selected and the corresponding pH denaturation curves were presented (see Figure 3). 

**Figure 4:**
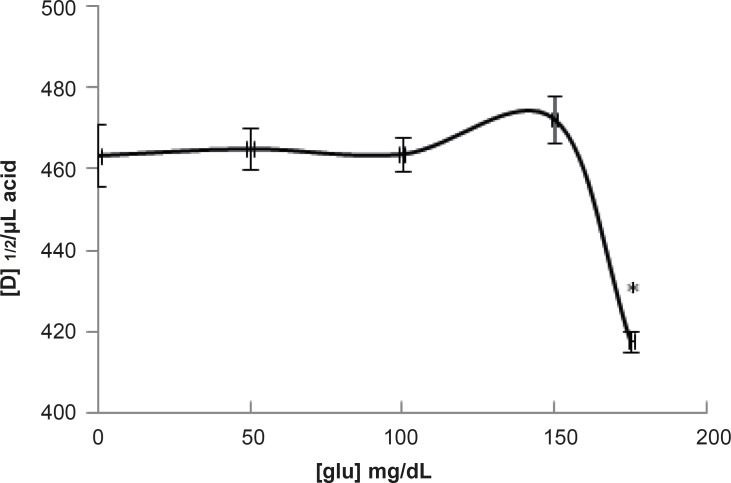
Representation of the amounts of [D]_1/2_ versus different concentrations of glucose (the amounts of [D]_1/2_ correspond to results that are tabulated in Table 1). * p-value < 0.0001

Then, the amounts of [D]_1/2_ for all of the curves accompanied by standard deviations (SD[D]_1/2_) were tabulated in Table 1. It seems that analysis of [D]_1/2_ can lead to differentiation of obtained results. Therefore, the amounts of [D]_1/2_ correspond to Table 1 are represented as Figure 4. 

**Figure 5:**
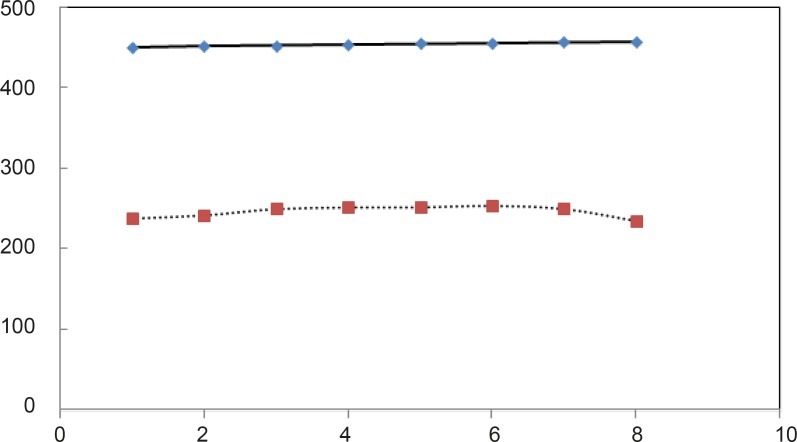
Times or repitations for albumin acid denaturation in preseance (…) and absence ( __ ) of fluoxetin at 37°C

For beter resolution, [D] _1/2_ parametr of acid denaturation albumin was calculated in the absence and presence of fluxetin 50 mM at 37°C with 8 times repitation and presented in Figure 5. ΔG^0^_H2O_ is introduced as stability parameter for proteins, so, the amounts of ΔG^0^_H2O_ are shown in Table 1 and illustrated as function of glucose concentration in Figure 5. 

**Figure 6:**
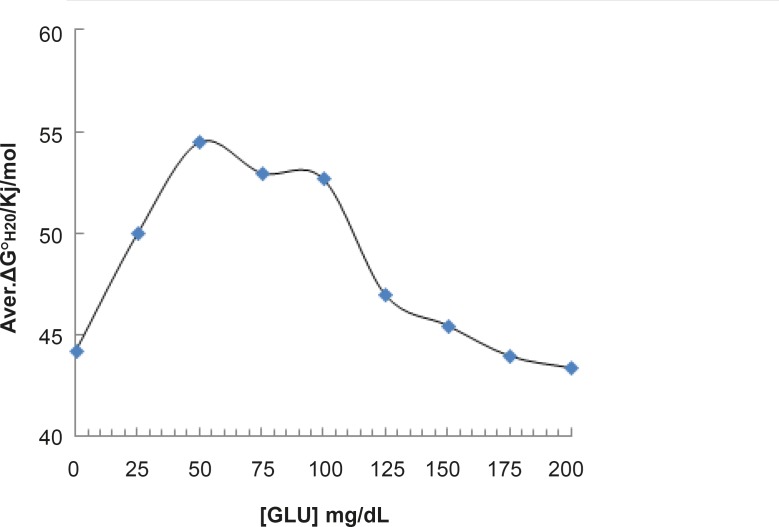
Illustration of the amounts of ΔG^0^_H2O_as function of different glucose concentration (correspond to Table 1).

Since the fluorescence is a sensitive method, the spectra of human serum albumin were provided in the presence of 100 and 175 mg/dL concentrations of glucose and shown in Figure 6.

**Figure 7:**
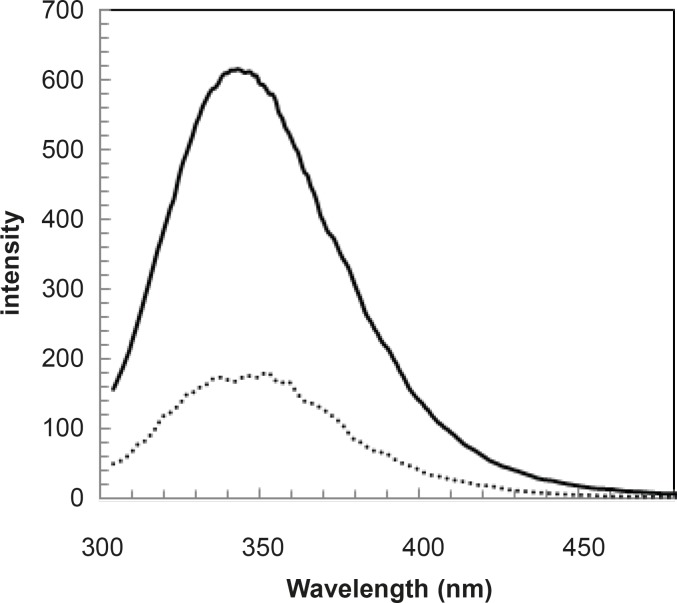
Fluorescence spectra of HSA in the presence of 1 g/L (…) and 1.75 g/L ( __ ) concentrations of glucose at 37°C in Tris buffer

**Table 1:** The amount of average of [D]_1/2_ and ΔG^0^_H2O_ for all experiments accompanied by standard deviation

**[Glucose]mg/dL Average of [D]**_1/2__/_ **μL acid**	**SD[D]**_1/2 /_ **μL acid**	**ΔG** ^0^_H2O_ **/ kJ/mol**
0	463.3 ± 7.6	44.2
50	465.0 ± 5.5	54.5
100	463.7 ± 4.0	52.7
150	472.3 ± 5.9	45.5
175	417.7 ± 2.5	43.4

## Discussion

Protein denaturation study is an important method for characterization of protein structure and function (1-3). This process is concerned grossly by environmental factors such as temperature, chemical components, *etc. *(2). Albumin is one of the most important proteins of body due to the variety of functions such as ligand binding capacity, hormones and a wide variety of drugs (4). Glucose concentration is constant in blood in normal condition but it can be changed via hypoglycemia and hyperglycemia (as diabetic condition), therefore, denaturation of human serum albumin is studied in this assay in the presence of various concentration of glucose. It is reported that normal concentration of glucose in blood is 100 mg/dL (7, 26) and for diabetic condition it is rises up to 400 mg/dL (26). It is important to know the effects of glucose on proteins function and structure in blood. Diabetic condition is accompanied with the occurrence of serious damages in body (27). Here, it is proposed that the structural and functional aspects of albumin are affected in diabetic condition. Different concentrations of glucose make variety conditions on body. It is reported that 175 mg/dL glucose concentration is a threshold of kidney activity for the beginning of excretion of glucose (28). There are many evidences that express 175 mg/dL glucose concentration induces several pathological conditions such as polyurea, polydipsia and polyphagia patients (29).

pH denaturation curves of albumin in the absence and presence of various glucose concentrations are shown in Figures 2 and 3. At first glance, it seems that the presence of glucose induces mild alteration in bufferic property of albumin (Figure 3). If protein denaturation process obeys the two-state theory, two important parameters such as ΔG^0^_H20_ and [D] _1/2_ can be calculated for denaturation process (21). [D]_1/2_ parameter is calculated and tabulated in Table 1. [D] _1/2_ is described as denaturant concentration that induces 50% of denaturation in protein molecular population and introduced as the protein resistance index against denaturant (24). As it is shown in Figure 4, [D] _1/2_ of albumin denaturation are the same in absence and presence of glucose concentrations by 150 mg/dL and have no statistical differences. So, we can explain that albumin probably doesn’t have any structural and functional alteration in absence and presence of these concentrations of glucose (0, 50, 100, 150 mg/dL). It is obvious that if the albumin structure and function change, the alteration cannot be detected by study of [D] _1/2_. Probably, the occurrence of negligible alteration in HSA structure and function in the presence of glucose concentration below 150 mg/dL is related to the physiological function of HSA. But as it is depicted in Figure 4, the amount of [D]_1/2_ albumin in the presence of 175 mg/dL glucose concentration shows gross difference (with consider statistical analysis) in compare to the others. There are many evidences about the effects of 175 mg/dL concentration of glucose on functional behavior of various tissues and organs in body, it is reported that liver, diabetic foot ulcer, connective tissue, retinopathy, glaucoma, auditory disorder (30-36). Here [D]_1/2_ can be consider as a reliable index for monitoring and understanding these alterations. [D]_1/2_ is discussed as the stability parameter and a suitable index correspond to the conformational change of protein in the presence of applied stress condition. Using [D]_1/2_ as a thermodynamic and specially physicochemical parameter, is suggested as a strong parameter in interpretation of protein denaturation study. Here [D]_1/2_ is used as a key point in solving a clinical problem. The previous studies indicate that fluoxetine effects on structural and functional aspects of albumin (17). It can be concluded that the presence of fluoxetine shall alter [D]_1/2_ of albumin in the acid denaturation process. As it is shown in Figure 5, the presence of fluoxetine not only has changed the amount of [D]_1/2_ but also attenuates it in the manor as the glucose effect in 175 mg/dL concentration. Binding of ligands to HSA sometimes induces changes in protein conformation and consequently its fluorescence. Probably, the fluorescence spectra of HSA indicated the gross conformational change of protein in the presence of 175 mg/dL glucose concentration related to 100 mg/dL glucose concentration. Finally, by considering all findings, it can be concluded that 175 mg/dL concentration of glucose is a clinical point for albumin structural and functional alteration. This finding is an important tool for interpreting the roll of glucose on diabetic disorder.
